# The Prevention and Treatment of Puerperal Fever*Read before the New York Academy of Medicine, December 6, 1883.

**Published:** 1884-02

**Authors:** T. Gaillard Thomas

**Affiliations:** Clinical Professor of Diseases of Women in the College of Physicians and Surgeons, New York


### Selections.

### The Prevention and Treatment of Puerperal Fever.*

By T. Gaillard Thomas, M. D.

Clinical Professor of Diseases of Women in the College of Physicians and Surgeons, New York.

Let us pause here and review the features in the condition of the puerperal woman which render her a prey to so many and dangerous disorders which spring up as consequences of utero-gestation and of parturition.

In the first place, her blood is in a condition of hyperinosis—that is to say, it contains a great excess of fibrin. If it be drawn by the lancet, it presents the buffy coat, upon which our forefathers laid so much stress, in the most marked degree; and from this arise two liabilities—first, a tendency in such blood to form thromboses in the heart and the blood-vessels, and, second, a tendency to prove a most prolific ground for the development of sepsis and zymosis. Measles, scarlatina, and varioloid, which

* Read before the New York Academy of Medicine, December 6, 1883.

give no very bad prognoses when they excite zymosis in the blood of the non-pregnant woman, commonly produce death when they act upon the blood of pregnancy.

Then the nervous system is in a plus state of sensitiveness and excitability, and influences which are very controllable in the non-puerperal state produce very evil results here. For example, an accumulation of urinary poisons in the blood produces convulsions; an untoward moral influence produces violent mania; and crude ingesta result in severe spasmodic affections in the alimentary canal, which, in the same woman when not pregnant, would scarcely have attracted attention.

The local conditions which result from parturition are even more striking. The uterus and other pelvic viscera are, at full term, as fully supplied by lymphatics and lymphatic glands as is shown in this diagram;* and the arteries, veins, nerves, and other tissues of that organ, the vagina, the uterine ligaments, and the peritonæum have all undergone a rapid physiological hypertrophy, which permits of an organ only three inches in length ascending so as to touch the ensiform cartilage.

The uterus about the 280th day of gestation contracts and expels the child; then the placenta and membranes; and then closes its emptied cavity, and rests. Let us suppose that in forty-eight hours after delivery a primipara dies of pneumonia, and that we are allowed to lay open the genital tract and examine it from the fundus uteri downward. Outside all looks well; the uterus is merely much larger than in the non-pregnant state. Within, it presents a very different appearance; the whole endometrium, covered over by the greyish, sloughy-looking decidua vera, presents all over its surface an unhealthy, unclean and diphtheritic look, although free from exudation. Here and there shreds of membrane, consisting of small portions of the decidua reflexa, which had become adherent, appear, partially detached and somewhat decomposed. At one point the large placental site is seen, raw, irregular, and covered over by minute

* Here Dr. Thomas showed diagrams, which we omit for lack of space.—Editor.

traces of the placenta and small blood-clots which close the mouths ot the uterine sinuses. The odor of the opened uterine cavity, the walls of which are thus covered, is disagreeable. The substances mentioned have for forty-eight hours been dislodging themselves and mingling with the pinkish fluid which pours like an unhealthy sweat from the placental site; constitute what is called the cleansings, or lochial discharge. Upon examining the cervix uteri, we find two or three small rents which pass through the mucous lining and involve to a varying depth the sub-lying parenchyma. In consequence of these injuries, and of absorption through them of the lochial discharge already mentioned, the cervix is swollen and œdematous. As we examine the vagina it will be found that the great distension impressed upon it by the head of the child in its passage to the vulva has in two or three places caused a superficial rupture of the mucous lining of this canal.

We now arrive at the vulva, and here we find several solutions of continuity which have been effected by the escaping head. The fourchette has been torn through, and this rent has extended through a small portion of the perinæum, and one or two small fissures have occurred in the mucous membrane covering over the ostium vaginæ.

Were we to take some of the lochial discharge from the vagina, after the atmosphere has acted upon it, and, abrading the inside of the finger with a lancet so that it bleeds slightly, apply this freely to the denuded surface, and allow it to become dry there, its irritating character would soon become evidenced by a burning sensation in the part, a smarting extending up the hand, and on the next day signs of a slight local inflammation, with a little lymphangitis, would be noticed. This would probably last only two or three days—merely long enough to demonstrate the fact that the fluid is an irritating one, but not sufficiently poisonous to cause erysipelas or severe angeioleucitis.

The natural history of the ordinary local results of human parturition is given in the foregoing sketch. In every case ot child-bearing the endometrium is thus encumbered and freed by a process of exfoliation and sloughing ; in every case the cervix, vaginal mucous membrane, perinæum, and vulva are, in varying degrees, lacerated ; and in every case the offensive fluid, called lochia, poisons these freshly made, unprotected wounds. And yet what are the usual results ? Recovery, uniformly, I might say universally, unless some unusual occurrence manifests itself to prevent this happy consummation ! Theorizing about the matter, one would suppose that the mortality resulting from such a state of things must be excessive. Here we have a number of recent wounds constantly and unavoidably bathed with a fluid made up of dead and decaying animal tissue in a woman whose blood and nerve states are, with reference to septic disease, like flax prepared for the spark, and who is exhausted by pain, anxiety, loss of blood, and deprivation of sleep. Can one point to any concatenation of circumstances better calculated to insure a bad result ? And yet the facts are these : only about one or two in every one hundred parturient women ordinarily die when properly cared for during labor, even in the public hospitals.*

Recovery, then, is the very general rule after normal parturition ; death the very rare exception. But now and then all this is changed. Some ferment or specific poison gains access to the genital canal and acts as rapidly and as decidedly as a little yeast added to dough. In the latter case, active and immediate fermentation affects the whole mass ; in the former a set of striking, alarming, and often fatal phenomena occur, which spread dismay through the lying-in chamber and give an entirely new complexion to the progress of the case. The fact that this unfortunate occurrence has taken place will usually announce itself to the attending physician in this way. He leaves his patient on the morning of the third day cheerful, happy, free from pain, with a pulse of 85, and a temperature of 99°. He is called to her in the latter part of that day and finds that she has had a slight, perhaps a scarcely perceptible, chill ; that some pelvic

* Tarnier, "Report of Maternite Hospital," Paris.

pain has followed it; that the lochia have ceased; that the milk which was just showing itself has disappeared; that a severe headache exists; that a look of indescribable anxiety has replaced the happy expression of the morning; that the pulse rate is 130 to the minute, and that the buccal temperature is 104°.

A poisonous element has by some method or other reached the genital tract, as fruitful a field for its activity as a mass of dough is for yeast, and the result is already manifesting itself. Let us suppose that the patient's medical attendant lays the flattering unction to his soul that all this is due to "malaria"; or that he soothes his troubled mind with the hope that it is "milk fever"; or that, recognizing the attack as one of "puerperal septicæmia," or "blood-poisoning of child bed," the first link in that terrible chain called puerperal fever, he relies upon medicines given by the mouth or rectum, what is usually the course shown as the natural one of the affection? Within a week, or thereabouts, for there is no rule as to this point, parenchymatous metritis, lymphangitis, lymphadenitis, phlebitis, cellulitis, or peritonitis will very probably develop itself, and what was originally merely a septicæmia will merge into one of these affections, and the patient will pass through the perils attendant upon whichever of these pathological states manifests itself as a consequence of the initial lesion.

Sometimes the septic disorder develops puerperal mania, while at other times a septic pleuritis, endocarditis, pneumonitis, pericarditis, or meningitis follows the systemic poisoning, the lymphatics emptying the deadly contents into the thoracic duct, and thus transferring them into the subclavian vein, as Lusk clearly points out. At other times the condition continues one of true and uncomplicated septicæmia to the end, death occurring from coma, or the patient succumbing to exhaustion from hyperpyrexia, which lasts for weeks. What was originally septicæmia, however, as a rule rarely remains so, but generally passes into some other disorder, and very generally into perito nitis, before a fatal termination occurs.

And now comes naturally the question, What is the pathology of that affection styled puerperal fever? An inquiry into the views which prevail among others would evidently require more time than I can possibly allot to it to-night; and yet I am desirous that my answer, even if very short, shall be so clear, succinct and simple as to convey perfectly the opinion which a practice of thirty years has impressed upon my own mind concerning a subject which has always deeply interested me, and in connection with which I have had abundant opportunity for study, both at the bedside and in the dead-house.

My observations have led me to adopt the views of those who believe that *puerperal fever is puerperal septicæmia*. It matters not whether it assume the form of metritis, phlebitis, cellulitis, peritonitis, or lymphangitis, the essence of the disorder is a poison, which is absorbed into the blood of the parturient woman through some solution of continuity, and which, in the appropriate soil of the puerperal condition, fructifies and produces the result known in its *ensemble* of pathological phenomena as puerperal fever. From my standpoint, the matter is well stated by Lusk * when he declares that " it has now passed beyond the domain of dispute that puerperal fever is an infectious disease, due, as a rule, to the septic inoculation of the wounds which result from the separation of the decidua and the passage of the child through the genital canal in the act of parturition."

As early as 1870, Hervieux, in his work on the diseases of child-bed, already alluded to, expressed himself upon this point in the following words: " Here I stand; if what I have said does not carry conviction of the truth of my doctrine, fuller explanation will fail to do so. I believe in the multiplicity of puerperal diseases. I believe in puerperal poisoning as the source of them. Here, in two words, my creed is presented."

In 1877 the Berlin Obstetrical Society appointed a committee on puerperal fever, consisting of Schroder, Lohlein, A. Martin Fasbender, and Boehr—men whose names are sufficient to com-

* " Midwifery," p. 608.

mand attention, even if their words fail to carry conviction. In its report this committee expresses its views thus:* "Under the names 'puerperal fever,' 'malignant child-bed fever,' are included a group of diseases occurring in child-bed which vary very greatly in their manifestations, but have this in common, that they are called into being by the absorption from the organs of generation of a material which gives rise to destructive inflammation and fever. There are, indeed, a number of substances, mainly composed of organic materials in a state of putrid decomposition, which, when brought into contact with an open wound, set up inflammation in it, which extends to the neighboring tissues; a further absorption by the lymphatics and blood-vessels leads to more extensive imflammation among neighboring and remote organs; and, when a large quantity is rapidly absorbed into the blood, a quickly fatal poisoning of the whole organism occurs. To surgeons the deadly effect of these materials upon wounds is only too well known, and the greatest advance, probably, which surgery has ever made consists in the so-called antiseptic method of treating wounds—that is, in the scrupulously exact removal of such materials from fresh wounds.

"Puerperal fever is indeed nothing else than the infecting of fresh wounds, such as are found in every newly delivered woman, with these destructive septic materials. Almost every woman, after labor, has small wounds on the external genital organs, which are caused by the passage of the child through this narrow opening, and in every newly delivered woman the inner surface of the uterus, from which the protecting membrane has been cast off with the ovum, presents a large wound surface. Thus, every newly delivered woman is liable to suffer from the dreaded infective wound diseases, which in persons wounded under other circumstances, are called pyæmia, septicæmia, wound-fever, blood-poisoning, purulent infection, etc., *so soon as suitable septic materials are brought into contact with the genital organs.*"

* "Report of Puerperal Fever Committee of Berlin Obstetrical Society," December 4, 1877, p. 9.

What is the nature of this subtle and deadly poison, which, entering like a ferment into the genital canal of the puerperal woman whose blood is hyperinosed, whose nerves are in a condition of hyperæsthesia, whose utero-placental vessels are partially open, whose cervix uteri, vagina and vulva are covered with fresh superficial wounds, and whose womb is pouring slowly forth a fluid composed of dead tissue, decomposed blood and recently exfoliated cells, gives rise to so much disturbance? What do we know of the poison? what is its natural history? what encourages its life? and what kills it, or cripples its activity?

Unfortunately, these questions cannot to-day be satisfactorily answered; but have such questions been any more satisfactorily answered with reference to scarlatina, measles, and varicella? German pathologists have proved that the presence of micrococci, more especially of the round bacteria, occurs so frequently in the pathological products of puerperal diseases as to lead to the conviction that they are important factors in reference to them; but this point, like many others connected with the influence of bacteria as morbific agents, is yet too unsettled for admission into a practical treatise like the present. * Inquiry into the matter is now being pushed with vigor in the laboratories of France and Germany, and we have, according to recent reports, a fair prospect of valuable and practical results.

But, even although we do not at present know the exact nature of the poison which proves the disturbing element in these cases, we surely know that some such toxic agent exists, and it behooves us to learn how to prevent its entranee into the genital tract, and how best to destroy its life or its activity if it should gain admission in spite of our care and watchfulness.

Whatever be the character of this agent, we know that there are *two, and only two*, methods by which it can reach the parturient tract and exert its baneful influence. First, it may be carried

* In reference to this part of our subject I would refer those interested to the chapter upon it by Professor Lusk, in his masterly book upon the "Science and Art of Midwifery."

to the vulva and into the vagina through the open orifice of that canal by the atmosphere, in which it floats as an impalpable substance; and, second, it may be carried to any part of the genital tract by the fingers of doctor or nurse; by towels or cloth laid against the vulva; by sponges used in washing; by instruments used in the delivery of the child, drawing of urine, or injecting the vagina; and from the bed-clothing and body-clothing of the patient which are in immediate contact with the sexual organs.

*Prophylactic measures which should be adopted in all midwifery cases, whether they occur in hospital or in private practice:*

1. The room in which the confinement is to take place should have the floor, walls, and furniture thoroughly washed with a ten-per-cent. solution of carbolic acid or mercuric bichloride, 1 to 1,000, and the bedstead and mattresses should be sponged with the same solution. Curtains, carpets and upholstered furniture should be dispensed with as far as possible.

2. The nurse and physician should take care that all their clothing, both under and upper, be clean and free from exposure to the effluvia of any septic affection. Should either of them have been exposed within a fortnight to the effluvia of such affections as scarlet fever, typhus, erysipelas, septicæmia, or the like, they should change every article of clothing and bathe the entire body, especially the hair and beard, with a reliable antiseptic solution; that which I prefer for this purpose is a saturated solution of boric acid.

3. As labor sets in, the nurse, having thoroughly washed her hands, cleaned her nails with a stiff nail-brush, and soaked them in antiseptic fluid, should administer to the patient a warm vaginal injection of antiseptic character; bathe the vulva and surrounding parts freely with the same; repeat this every four hours during labor; and keep a napkin, wrung out of the warm antiseptic fluid, over the genital organs until the birth of the child.

4. Before assuming the functions of their respective offices at the moment of labor, both doctor and nurse should wash the hands thoroughly with soap and water, *scrub* the nails with a stiff nail-brush, and soak the hands for several minutes in a bichloride solution, 1 to 1,000.

5. The first two stages of the labor having been accomplished, the third stage should be efficiently produced; all portions of placenta and membranes removed; and ergot administered, in moderate doses, three times a day, and kept up for at least a week, for the complete closure of the uterine cavity, expulsion of clots, and occlusion of the utero-placental vessels.

6. The doctor, taking nothing for granted, not satisfying himself with a vague report of the nurse, should, at the conclusion of the labor, carefully examine the vulva of the patient. If the perinæum be lacerated, it should be closed at once by suture, to shut up this avenue to septic absorption; and, should slight solutions of continuity be found in the labia or the vulvar extremity of the vagina, these should be dried by pressure of a linen cloth, touched with equal parts of sol. ferri persulph. and carbolic acid, again dried thoroughly by pressure with the cloth, and then painted over with gutta-percha collodion. If this be thoroughly done, absorption will be prevented at these points for at least three or four days, when the application may be repeated.

7. In six or eight hours after the labor, when the patient has rested, the vagina should be syringed out with an antiseptic solution, and a suppository of cocoa butter, containing from three to five grains of iodoform, should be placed within it, under the os uteri. A syringe with intermittent jet should be used, which will wash away with gentle force all blood-clots, and reliance should not be placed upon the feeble drip of the fountain syringe, the advantages of which are, I think, entirely theoretical.

8. These vaginal injections and suppositories should, in cases of normal labor, be repeated every eight hours; in cases of difficult or instrumental labors, twice as often; and they should be kept up for at least ten days, the nurse observing to the last the precaution already mentioned of washing her hands before every approach to the genital tract of the patient.

9. When catheterization becomes necessary, it is safer to employ a new gum-elastic catheter, which before use should be thoroughly immersed in antiseptic fluid, and which should be destroyed at the conclusion of the case, rather than to trust to the nurse's cleansing of an old silver instrument which bears within it the register of a list of cases of septicæmia in which she has employed it during the past two or three years. *It is a very commom and very bad habit for nurses to own silver catheters, which they carry about with them from case to case of midwifery.*

10. Last, but by no means least, let the physician inform himself by personal observation as to the competency of the nurse to syringe out the vagina thoroughly, to place the antiseptic suppositories just where they should be, and to use the catheter without injury to the patient. Neglect of this precaution has frequently resulted in leaving a fœtid upper segment of the vagina entirely unwashed, while the antiseptic stream was limited to the lower third of the canal.

Before leaving this part of my subject, let me, in the strongest manner, record my protest against the use of intra-uterine injections as a prophylactic measure, except after very severe operations within the uterine cavity, which render the occurrence of septicæmia almost certain. As a preventive measure, to be uniformly adopted, I look upon it as to the last degree rash and reprehensible. The use of a dangerous remedy in combating the results of a dangerous disorder is always admissable, but a resort to a hazardous procedure for a condition in connection with which danger has not shown itself, and may not do so, should be viewed in quite a contrary light.

